# Impact of transitioning to virtual delivery of a cardiovascular health improvement program for Latinos during the COVID-19 pandemic

**DOI:** 10.1186/s12889-022-14291-6

**Published:** 2022-10-18

**Authors:** Amelia Iglesias, Ashley Ambrose, Stephanie Coronel-Mockler, Kristin Kilbourn, Marc P. Bonaca, Raymond O. Estacio, Mori J. Krantz

**Affiliations:** 1 University of Colorado School of Public Health, Aurora, CO USA; 2 CPC Community Health, Aurora, CO USA; 3 Department of Medicine, Denver Health and Hospital Authority, Denver, CO USA; 4 Department of Psychology, University of Colorado, Denver, CO USA; 5 Division of Cardiology, University of Colorado School of Medicine, Aurora, CO USA

**Keywords:** Latino health, Community health, Cardiovascular disease, Health promotion, COVID-19

## Abstract

**Background:**

Community Heart Health Actions for Latinos at Risk (CHARLAR) is a promotora-led cardiovascular disease (CVD) risk-reduction program for socio-demographically disadvantaged Latinos and consists of 11 skill-building sessions. The COVID-19 pandemic has led to worsening health status in U.S. adults and necessitated transition to virtual implementation of the CHARLAR program.

**Methods:**

A mixed-methods approach was used to evaluate virtual delivery of CHARLAR. Changes in health behaviors were assessed through a pre/post program survey. Results from virtual and historical (in-person delivery) were compared. Key informant interviews were conducted with promotoras and randomly selected participants and then coded and analyzed using a thematic approach.

**Results:**

An increase in days of exercise per week (+ 1.52), daily servings of fruit (+ 0.60) and vegetables (+ 0.56), and self-reported general health (+ 0.38), were observed in the virtual cohort [all p < 0.05]. A numeric decrease in PHQ-8 (-1.07 p = 0.067) was also noted. The historical cohort showed similar improvements from baseline in days of exercise per week (+ 0.91), daily servings of fruit (+ 0.244) and vegetables (+ 0.282), and PHQ-8 (-1.89) [all p < 0.05]. Qualitative interviews revealed that the online format provided valuable tools supporting positive behavior change. Despite initial discomfort and technical challenges, promotoras and participants adapted and deepened valued relationships through additional virtual support.

**Conclusion:**

Improved health behaviors and CVD risk factors were successfully maintained through virtual delivery of the CHARLAR program. Optimization of virtual health programs like CHARLAR has the potential to increase reach and improve CVD risk among Latinos.

**Supplementary Information:**

The online version contains supplementary material available at 10.1186/s12889-022-14291-6.

## Background

In the United States, Latinos are one of the fastest-growing racial/ethnic minority populations, constituting 18.3% of the population by 2019 estimates [1, 2]. Cardiovascular disease (CVD) and diabetes are among the leading causes of death within the U.S. [2] and a majority of Latinos possess at least one major CVD risk factor [3–5]. Moreover, Latinos are 50% more likely to develop diabetes in their lifetime compared to non-Hispanic whites [2].

To effectively address CVD and diabetes risk in the Latino population, interventions commonly utilize community health workers or health promoters, commonly referred to as promotoras [6]. Promotoras provide education and support to Latino communities to help improve health outcomes including CVD and diabetes risk factors [2, 7]. As members of the Latino community, they possess essential cultural knowledge and do not have to overcome language barriers, making them effective health educators and advocates for the Latino community [7]. Promotoras have been used in numerous CVD interventions among Latinos [8–10], and Community Heart Health Actions for Latinos at Risk (CHARLAR) is specifically tailored to Latinos in Colorado, including monolingual Spanish speakers.

CHARLAR is an 11-week evidence-based health promotion program which provides health screenings and referrals to medical and behavioral health care. CHARLAR promotoras also teach classes and foster a sense of community. CHARLAR leads to significant improvements in major modifiable CVD risk factors [11], however, this was achieved through face-to-face implementation in trusted community settings such as churches where direct physician counseling was provided. As a result of the COVID-19 pandemic, CHARLAR transitioned from in-person classes to an online format, significantly altering program implementation. This manuscript reports the acceptability of virtual delivery of the CHARLAR program and its impact on modifiable CVD risk factors.

## Methods

In March 2020, starting at week six of the 11-week cohort, promotoras implemented classes online through Zoom® (San Jose, CA) and Facebook Live® (Menlo Park, CA). This study evaluated how the online transition during the COVID-19 pandemic impacted program outcomes and promotora and participant experience of the program. The online format, implemented in half of the original two-hour time period, included 30 minutes of recorded content using the original curriculum PowerPoint slides, and 30 minutes of group discussion, activities, and review of handouts. The curriculum PowerPoint slides were adapted to be delivered in a presentation format. Slides with repetitive information that were originally included to foster increased understanding were not reviewed in-depth given time constraints. The group discussion and activities, originally included in the curriculum PowerPoint slides, were instead implemented following the presentation and adapted to accommodate virtual instruction. With the use of online tools like screenshare and breakout groups in Zoom® (San Jose, CA), the original discussions and groups were largely maintained. In addition, promotoras called each participant prior to the first class to ensure they understood how to connect to the virtual classes. Additional follow-ups were provided after each class to ensure participants had the support needed to participate in the virtual class and access community health resources.

In order to understand the impact of shifting to a virtual format, we sought to understand: acceptability of the core components of the remote CHARLAR program, the facilitators and barriers to implementation, and how the switch impacted promotoras’ ability to sustain participant rapport and deliver the curriculum. We also assessed how the virtual format impacted participants’ ability to learn, improve health behaviors and connectedness, and achieve CVD and behavioral health risk factor improvements.

The evaluation was mixed methods. Quantitative data were derived from pre-survey (n = 105) and post-survey (n = 81) data from the virtual cohort and compared to pre-survey (n = 184) and post-survey (n = 123) data from a historical cohort delivered in-person the year prior to the pandemic. Survey data contained items related to fruit and vegetable consumption, sugar-sweetened beverage intake, exercise, general health, and mental health. Quantitative survey items, scales and scores relating to food and beverage consumption, exercise and general health were derived from the CDC’s validated Behavioral Risk Factor Surveillance System (BRFSS) Questionnaire. The seven-item Generalized Anxiety Disorder Scale (GAD-7) questionnaire was utilized given its reliability and validity in public health settings [12]. Similarly, the eight-item Patient Health Questionnaire depression scale (PHQ-8) was used to assess for depression as it has been validated to measure the severity of depressive disorders in population-based studies [13].  For the virtual cohort only baseline biometric measurements were obtained since it occurred prior to COVID-19 social distancing restrictions in Denver. A paired t-test was conducted to assess changes in weekly exercise, self-reported general health status using an absolute five-category ranking (poor to excellent), daily fruit and vegetable consumption, daily sugary beverage intake, and measures of anxiety and depression. Mean changes between virtual and historical (in-person cohorts) were compared. Statistical analysis was performed using SPSS (version 27.0, Armonk, New York) software. Attendance was tracked in Excel and proportional attendance between virtual and historic cohorts was compared using a Chi-Square Test. For all comparisons, a p-value < 0.05 was considered to represent statistical significance.

Qualitative data was collected via 25- 45-minute, semi-structured interviews with promotoras (n = 3) and participants (n = 6) by trained program staff with scripted guides. Separate interview guides were created for interviews with participants and promotoras. The interview guide for participants was translated from English to Spanish, and the participant interviews were conducted with a translator providing real-time translation for the English-speaking interviewer and Spanish speaking participants. Interviews, with the interviewer, participant and translator present, were conducted via phone and Zoom® (San Jose, CA). Interviews were audio-recorded with participant permission and transcribed verbatim. Respondent validity was obtained through member checks where promotoras confirmed accuracy and resonance of the data with their experiences. To ensure confirmability, only findings that were confirmed through multiple accounts were included in the qualitative findings. Nvivo Software (10.0, Melbourne, Australia) was used to conduct a thematic analysis of the qualitative data.

## Results

As shown in Table 1, the majority of participants in both cohorts were women (88.8%) in their 40s (39.1%). Most identified Mexico as a country of origin (83.4%) and preferred to speak Spanish (95.7%). The majority lived in the U.S. for more than 10 years (83.4%). 48% of participants had a family income of <$20,000 per year.

**Table 1:** Demographic and clinical characteristics of CHARLAR program participants

Demographics	Virtual Cohort N = 105 n (%)	Historical In-person Cohort N = 197 n (%)
**Age Group, years**		
20–29	7 (6.7)	10 (5.0)
30–39	18 (17.1)	37 (18.8)
40–49	44 (41.9)	74 (37.6)
50–59	24 (22.9)	54 (27.4)
60+	12 (11.4)	20 (10.2)
**Sex**		
Male	16 (15.2)	30 (15.2)
Female	89 (84.8)	167 (84.8)
**Preferred Language**		
English	2 (1.9)	2 (1.0)
Spanish	100 (95.2)	189 (95.9)
**Education**		
Less than High School	65 (61.9)	84 (42.6)
High School or Higher	32 (30.5)	69 (35.0)
**Country of Origin**		
United States	5 (4.8)	7 (3.6)
Mexico	93 (88.6)	159 (80.7)
Other	5 (4.8)	10 (5.1)
**Length of time in the United States**		
0–10 years	8 (7.6)	18 (9.2)
>10 years	94 (89.5)	158 (80.2)
**Family Income**		
≥$20,000/ year	51 (48.5)	62 (31.5)
<$20,000/ year	48 (45.7)	96 (48.8)
**Employment (p = 0.002)**		
Employed	37 (35.2)	73 (37.1)
Unemployed	18 (17.1)	18 (9.1)
Retired	5 (4.8)	1 (0.5)
**Risk Factors**		
Smoker	5 (4.8)	10 (5.1)
Hypertension	12 (11.5)	36 (18.3)
Diabetes	17 (16.2)	18 (9.1)
High Cholesterol	14 (12.4)	32 (16.2)
Heart Disease (p = 0.02)	5 (4.8)	1 (0.5)

**Fig. 1:**
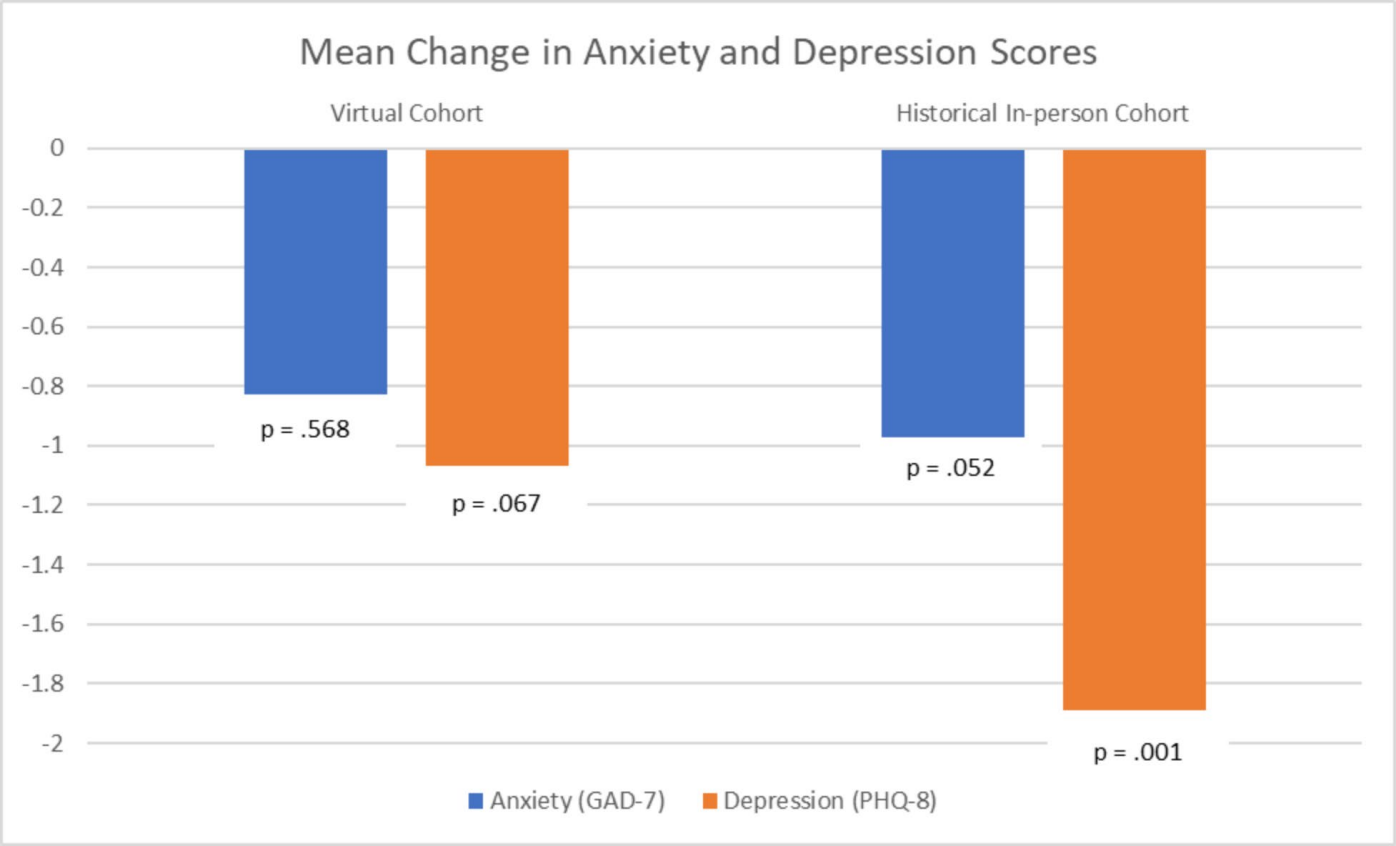
Mean Change in Anxiety and Depression Scores. Although not statistically significant, the change from baseline (pre-program) in both anxiety and depression scores were directionally similar for both in-person and virtual program delivery. The Y-axis is change in mean points for both anxiety (blue) and depression (red) scores. *GAD = Generalized Anxiety Disorder 7 item inventory. PHQ = Patient Health Questionnaire 8 item inventory.

The response rate for the pre-survey was 100%. Examination of the pre- and post-survey data for the virtual CHARLAR cohort show significant improvements in days of exercise per week (+ 1.52 days), daily fruit consumption (+ 1.35 servings), daily vegetable consumption (+ 0.56 servings), and self-reported general health (+ 0.38) (all p < 0.05). There was no significant change in daily exercise time and sugar sweetened beverage consumption for the virtual cohort. In comparison, the historical cohort also exhibited increase in days of exercise per week (+ 0.91 days), exercise time (+ 7.78 min per day), daily fruit consumption (+ 0.244 servings), and daily vegetable consumption (+ 0.282 servings) (all p < 0.05). There was no significant change in self-reported general health and daily sugar sweetened beverage consumption for the historical cohort (Table 2). Additionally, within the virtual cohort, there was a numeric decrease in anxiety (-0.83, p = 0.568) and depression (-1.07, p = 0.067) scores (Fig.1). In the historical cohort, there was a significant decrease in depression scores (-1.89). Changes in anxiety scores (-0.972) were not significant (p = 0.052).

**Table 2:** Mental and physical health outcomes among virtual and historical in-person CHARLAR program participants

	Virtual Cohort				Historical In-person Cohort			
**Variable**	**Mean Pre-Survey**	**Mean Post-Survey**	**Mean Change**	**P-value**	**Mean Pre-Survey**	**Mean Post-Survey**	**Mean Change**	**P-value**
Anxiety (GAD-7)	3.9	3.07	− 0.83	0.568	4.81	3.83	− 0.972	0.052
Depression (PHQ-8)	3.75	2.68	-1.07	0.067	5.28*	3.39*	-1.89*	0.001*
Exercise Days	1.90*	3.42*	1.52*	0.001*	2.08*	2.99*	0.91*	0.001*
Exercise Time (min/day)	25.97	30.00	4.03	0.192	30.05*	37.83*	7.78*	0.015*
General Health (scale of 1–5)	2.44*	2.82*	0.38	0.000*	2.56	2.67	0.11	0.754
Fruit Consumption (per day)	1.468*	2.820*	1.352*	0.000*	1.2356*	1.4792*	0.244*	0.004*
Vegetable Consumption (per day)	1.228*	1.790*	0.562*	0.000*	1.0187*	1.3011*	0.282*	0.005*
Sugar-Sweetened Beverage (per day)	1.093	1.316	0.223	0.213	0.9914	0.8594	0.131	0.256

^Abbreviations: GAD = Generalized Anxiety Disorder, PHQ = Patient Health Questionnaire^

^Statistically significant changes starred (*)^

^Statistical tests used: paired samples t−test^

Analysis of attendance data from virtual CHARLAR classes show that on average, participants attended four out of six (67%) of the virtual CHARLAR sessions, compared to five out of the last six classes (78%) of the historical in-person cohort. Of the 11 classes overall, mean participant attendance was eight of 11 sessions (73%) in the cohort with virtual classes compared to nine of 11 sessions (82%) in the historical cohort (p = 0.613).

The participant interviews revealed more detailed information about the impact of the virtually delivered CHARLAR program. A total of five participant themes were identified: (1) improving health habits, (2) mental health, (3) delivery challenges, (4) adaptability and flexibility, and (5) interpersonal connection. Quotes supporting these themes are provided, with edits for clarification indicated by square brackets.

### Improving Health Habits

When asked how the CHARLAR program impacted their health, participants reported that through the program they learned new information about CVD and diabetes, their health status, and how diet and exercise can impact health. This information empowered many participants to implement lifestyle changes focused on improving CVD risk factors. Participant 2 states:

"It has helped to motivate me to eat more healthy and to do exercise."

Participant 3 also shared how CHARLAR helped her to change her habits in a manageable way. She stated:

"Yes. Um, I feel like the class actually has helped me like learn how to change my habits on how to eat and stuff and what’s bad and what’s not bad and little by little I’m getting things into my head and sticking into the goals that okay, I’m gonna walk 20 minutes every day you know, for the whole week."

### Mental Health

When asked how the online program impacted both understanding and behaviors related to mental health, some participants noted the program helped them to learn more about anxiety and depression. Participant 6 shared:

"It was really good for [my] mental health, because [I] started doing exercises for relaxation and breathing, and [I] also learned more about anxiety and depression. And so that was helpful."

Another participant shared that CHARLAR was beneficial to her mental health as weekly goal setting helped her focus on gratitude and how positive thinking can impact her physical health. Still, others appreciated the extra support in general. Participant 2 shared how being asked about mental health allowed her to see how it could impact her home life:

"They asked about my mental health like if I had fears, if I had stress, if we were well and how we were doing and I don’t always associate those things with home life and so it opened my mind to that."

### Delivery Challenges

The qualitative interviews captured some of the challenges of pivoting to a new mode of delivery. Although the online classes broadcasted over Zoom and Facebook Live used the original curriculum, the majority of the educational content was condensed into a 30-minute recorded video, which was followed by 30-minute group discussions. Pivoting to a remote learning platform created several technological challenges. Previously, participants and promotoras had varied information technology experience and had challenges and apprehension downloading and operating the online software. Promotora 3 states:

“So, my apprehension was with me, me personally, I just wasn’t comfortable. I’ve been doing classes and teaching for over 25–30 years and so this was very different for me. I need that personal one-on-one contact. And so, it was hard for me to kind of just accept it and go with the flow, but it was better for me to learn it and help explain it than them not getting the class at all.”

Despite the difficulties, over time it became less daunting, and even had the unintended benefit of helping people become more comfortable around technology. Promotora 3 explains how, with time, using technology became more manageable:

"Like I said [using technology] was practice, it got easier. And it got easier to explain as well, because if I didn’t know what were some of the chat and all these other things… I was able to explain it the best way that I knew how and as simple as I knew how to explain it so that way, folks could get the best, you know, the best experience."

Promotoras suggested that the CHARLAR program offer more training for promotoras in facilitating discussions through virtual formats. Additional information to improve the virtual delivery of the program could be incorporated into future trainings.

Irrespective of the inherent loss of face-to-face connection, all of the promotoras and participants recognized that technology challenges were to be expected and they expressed general acceptance of the platform given the inherent necessity. However, all promotoras and participants expressed a preference for in-person classes. Across all nine interviews there were a total of 13 references from participants and promotoras expressing a preference for in-person classes.

### Adaptability and Flexibility

Promotoras were flexible and willing to learn to implement virtual CHARLAR and adapt to new situations. This greatly benefited the program, as it allowed for adaptation as promotoras, and program staff learned how to improve the online format. Initially, participants were watching the main video content independently and then coming on to video chat platforms for discussion as a larger group. When promotoras realized that discussion was difficult with such a large group, they quickly pivoted to smaller discussion groups. Promotora 2 explains how small groups impacted participants:

“Yeah, it’s easy and I think the participants they feel more comfortable to speak with three or four people than more than 20.”

Furthermore, promotoras and participants adjusted to the new technology with practice. Some participants were able to receive help from children or family members who were more familiar with technology. Participant 3 explained how her son was able to assist her when she needed help operating Zoom. She shared:

“At first, it was hard. I couldn’t figure it out. I just didn’t know how to be unmuted. I didn’t know. I was like messing with it. It was hard. I was asking my teenager. I’m like help me with this. And I’m not that old. But I don’t use this. I don’t do this, so I’m like, 'Help me. I don’t know how to do this.' It was a little difficult.”

Promotoras have also expressed interest in making further adaptations to achieve better outcomes. When asked about suggested improvements to virtual program delivery, promotoras suggested experimenting with longer class times.

### Interpersonal Connection

Transitioning to a virtual platform had effects on personal connectedness. Overall, participants and promotoras expressed initial challenges developing an “in-depth connection” with the participants. Promotora 2 explains these challenges:

"A lot of times Hispanics will speak with their eyes or with their faces and it can be a little bit difficult to connect with people when you’re having conversations with them online. So, sometimes those conversations can seem a little bit more cold, and there’s not as much of that humanity there that you would have in person."

While technology initially served as a barrier to connectedness, the promotoras adapted through additional follow-up calls. In addition to the videos and small group discussions, the promotoras checked-in with participants after each class. These weekly telephone check-in sessions allowed the promotoras to answer questions and ensure that the participants were able to access the class. The calls had the added function of helping to sustain rapport between promotoras and participants. Furthermore, small groups combined with video and follow-up calls have allowed CHALAR to reinforce information from each session via various modalities. Promotora 1 states:

"We feel that between the videos, that way we have all the integrity of the program, so that we really are passing the message in the way that is designed and the small groups and the calls we are the, you know, having all the components that we needed for CHARLAR."

## Discussion

This study demonstrates that transition to a virtual platform maintained effectiveness of the CHARLAR intervention, improving health among a minority population. First, we observed even larger improvements for health promoting behaviors such as exercise, fruit and vegetable consumption among the virtual CHARLAR cohort compared to the in-person cohort. Second, we observed a numeric reduction in depression score via the virtual platform, though to a lesser extent relative to the historical in-person cohort. Third, acceptability for both stakeholder groups (promotora and participant) was demonstrated in the qualitative interviews. What is novel about the current study is that despite the CHARLAR participant preference for face-to-face engagement, we observed surprisingly similar magnitude improvements in health behaviors with the virtual platform as compared with a historical cohort one year prior. While numerically lower, virtual attendance was not statistically significantly different between cohorts. This has important public health implications for CVD and mental health intervention dissemination during future COVID-19 pandemic surges or other states of emergency. Moreover, it has the potential to improve reach and scalability among Latinos, which are the fastest growing minority population in the U.S. [1]. This has potential utility since Latino internet usage has increased from 64% in 2009 to 84% in 2015 [14], making this a tenable public health platform. While each community health program is unique and maintenance of program effectiveness and viability across virtual platforms will vary, emerging evidence supports the importance of leveraging available infrastructure and technology to amplify virtual community health promotion efforts during the COVID pandemic [16]. Participant outcomes from this study demonstrate the importance of continuing the program while in-person classes were not permissible according to CDC guidelines [15].

On an international level, the COVID-19 pandemic has produced worsening glycemic, blood pressure and lipid control due to decreases in physical activity and increases in sugary food and snack consumption during the first two months of the pandemic [17–22]. The CHARLAR results, coupled with participants’ reported experiences of what they learned in classes, demonstrates that health information can be conveyed via online classes effectively, despite the stressors and challenges posed by the COVID-19 pandemic, and that the virtual implementation of the CHARLAR program was beneficial to participants.

Importantly, mental health outcomes also seemed to be ameliorated despite the negative population trends towards worsening anxiety and depression during COVID-19 [17, 23, 30]. Although not statistically significant, there were numeric reductions in anxiety and depression scores pre- and post-intervention, despite significant mental health stressors faced by families at the time of stay-at-home orders. Pandemic-related stress is associated with both anxiety and depression [30], and since April 2020, the U.S. has seen an increase in anxiety and depression [17, 23], with a three-fold increase in depression symptom prevalence during the COVID-19 pandemic [31]. Notably, Hispanics reported higher prevalence of anxiety and depressive disorders compared with non-Hispanic whites or Asians in the U.S. during the pandemic [30]. While non-statistically significant improvements were demonstrated in the quantitative data, mental health was not addressed explicitly in most of the qualitative interviews. These findings could stem from cultural perceptions of mental health among Latinos. Often mental health has negative connotations among Latinos, or they are seen as stemming from factors outside of one’s control [23]. Stress, anxiety, and loneliness stemming from the pandemic might have caused the behavioral health education component of CHARLAR to be less impactful. However, the involvement in the program could have served as a protective factor, facilitating relative behavioral health stability.

Community health workers (CHWs) have made significant contributions to promoting health equity [25] and improving health for chronic disease [26]. Their shared identity within community is central to galvanizing participant trust and supporting behavior change. Recent evidence emphasizes the CHW role in transitioning health programs to remote platforms during the pandemic [27, 28, 29]. The current qualitative aspect of our study illustrates the importance of the promotora-participant relationship in transitioning a program online, as most participants expressed gratitude for ongoing health support. Prior studies also suggest that CHWs gained resiliency in their work during the pandemic through creative thinking and maintaining positivity and focus [27, 28]. These findings mirror the current study where promotoras described the satisfaction gained by overcoming barriers in technology to maintain rapport and support for their participants.

Established barriers to CHW-led remote work during COVID [27] were recapitulated in our study, where promotoras initially felt that transitioning to a remote format hindered the provision of services and participant relationships. But as demonstrated previously [24], there are unexpected benefits for engaging under-resourced communities, such as expanding access to participation by allowing flexible attendance options and eliminating barriers to transportation and childcare. The CHARLAR transition to a virtual model further demonstrates these benefits as attendance was maintained, albeit at a lower level, and participants felt they were still able to improve their health behaviors. Transitioning to a virtual model can pose unexpected benefits to promotoras, including access to new technologies and resources, development of new skills and personal change [27]. Through our qualitative interviews, CHARLAR promotoras indicated greater confidence in their ability to navigate change, further strengthening their program engagement and sense of value to the community.

We acknowledge several limitations with this study. First, the absence of biometric measures in the post-program survey. COVID-19 restrictions [15] prevented promotoras and participants from conducting in-person screenings, which included height, weight, body mass index, blood pressure, cholesterol and glucose levels. This limits risk-factor correlation with lifestyle improvements. Language concordance was ensured by having a translator present during all interviews, and interview guides were translated into Spanish prior to conducting interviews to ensure clarity of questions. Translation fidelity was ensured by having an additional translator confirm accuracy of the translation after listening to the interview audio recordings. Nonetheless, absence of language congruent face-to-face communication could impact participants’ responses. While comparisons between cohorts are less robust than a prospective randomized design, this was not possible during the pandemic. However, the lack of statistically significant differences in baseline characteristics between cohorts allow for reasonable inferences regarding virtual program effectiveness. Another limitation is confounding effects from the COVID-19 pandemic which could alter pre- and post-survey measures preventing ascertainment of improvements in global (10-year) CVD risk score. However, the absence of worsening anxiety and depression coupled with improvements in healthy lifestyles suggests that the program retained its effectiveness for “whole-person health” despite the pandemic. Finally, the current study has transferability across the U.S. Latino population but is relevant primarily to the monolingual population.

## Conclusion

Overall, the change to a virtual delivery for CHARLAR significantly impacted promotoras’ and participants’ experience of the program. However, it did not prevent CHARLAR from providing essential CVD and diabetes education to participants. Despite the challenges, the virtual program achieved significant improvements in exercise, diet, overall health, and behavioral health comparable to the historical (in-person) cohort. These findings come at a critical time and have the potential to shape future implementation of CHARLAR and other promotora-led community health programs. Specifically, this may inform optimization of virtual program delivery and provide flexibility and opportunities to expand outside of Denver, benefiting other groups in need. Most importantly, understanding CHARLAR’s experience can provide information and examples to other programs attempting to implement virtual delivery of public health programs during and after the COVID-19 pandemic.

## Electronic supplementary material

Below is the link to the electronic supplementary material.

Supplementary Material 1

## Data Availability

The datasets and interview transcripts used and/or analyzed during the current study can be accessed using the following link: https://cpcmed.box.com/s/4ls6hejafyxn1jeskmhch7oevvnit4tn.

## Abbreviations

CHARLAR: Community Heart Health Actions for Latinos at Risk
CHW: Community Health Worker
CVD: Cardiovascular Disease
GAD-7: Generalized Anxiety Disorder Scale
PHQ-8: Patient Health Questionnaire Depression Scale
