# Supplementary material for: Impact of transitioning to virtual delivery of a cardiovascular health improvement program for Latinos during the COVID-19 pandemic
Source: BMC Public Health. 2022 Oct 18;22:1935. doi: 10.1186/s12889-022-14291-6 (PMC9579581; doi:10.1186/s12889-022-14291-6)
Supplement: Supplementary file 1 — Supplementary Material 1 [file 12889_2022_14291_MOESM1_ESM.docx]

**Community Heart Health Actions for Latinos at Risk**

**Documento de Consentimiento Para Los Estudios**

**PRE-Programa**

Usted está a punto de participar en un chequeo de salud. Las pruebas de salud son para medir el colesterol, la presión arterial, el azúcar en la sangre, el peso y la altura. También le haremos algunas preguntas personales, por ejemplo, dónde vive, qué come y qué tan activo es usted. Para el examen de colesterol y de azúcar en la sangre, necesitamos pinchar (picar) su dedo para conseguir una gota de sangre. Esto le puede doler un poco, como una cortada con papel. Su dedo puede estar adolorido por uno o dos días. Usted pudiera desarrollar una infección pero esto no sucede usualmente.

Al dar su consentimiento de participación, usted está de acuerdo en hacerse responsable de cualquier resultado obtenido de los exámenes. CPC y VUELA for Health son las organizaciones que patrocinan este evento y vamos a hacer todo lo que podamos para tener cuidado y no lastimarlo de ninguna manera. Pero si alguna cosa sale mal, usted está de acuerdo en no hacer responsable a CPC ni VUELA for Health de ningún problema que se presente por estos estudios. Usted está de acuerdo en hacerse responsable de cualquier lesión que usted pudiera tener como resultado de los estudios, aunque sea causada por CPC, VUELA for Health, las organizaciones participantes, los profesionales de salud o voluntarios del evento.

Al firmar esta forma de consentimiento, usted entiende que:

1. Este chequeo de salud no será utilizado para diagnosticar una enfermedad. Las pruebas son para descubrir si hay alguna prueba adicional que quizás se necesita hacer con un proveedor médico.
2. Toda la información que nos proporcione la mantendremos confidencial, a la medida de nuestra capacidad.
3. Un profesional de salud o un promotor de salud le explicará a usted los resultados de las pruebas y puede recomendarle otras acciones que usted debe tomar.
4. Los resultados de las pruebas pueden ser compartidas con su proveedor de salud o doctor y usted autoriza esto al firmar este documento de consentimiento. Sin embargo, es su responsabilidad personal conseguir seguimiento con su doctor o proveedor de salud. CPC, así como las organizaciones y los profesionales de salud que participan no aceptan ninguna responsabilidad para dar seguimiento a su cuidado.
5. Si usted no tiene un proveedor de salud o doctor, un representante de CHARLAR puede ayudarle a localizar uno.
6. Usted no sabe alguna razón porque no debe participar.

Si elige discutir su información con un médico en el evento de CHARLAR, la discusión puede tener lugar en un espacio público. CPC y VUELA for Health no proporcionarán los resultados de sus exámenes o de la encuesta a nadie. Sus resultados de las pruebas se pueden utilizar para escribir un informe, pero los resultados no tendrán su nombre.

**Nombre del participante (letra imprenta):** ______________________________________

**Firma del participante:** ______________________________________**Fecha:**___________________________

(Mes escrito)

**Community Heart Health Actions for Latinos at Risk**

**Pre-Program Health Screening**

**Fecha de Hoy:**

(Mes Escrito)

**Apellido** **Nombre**

**Dirección**

**Cuidad** **Estado**  **Código Postal**

**Número Principal** **Casa / Trabajo / Celular**

**Otro Teléfono** **Casa/ Trabajo / Celular /Familiar**

¿**Prefieres recibir recordatorios de clase por mensajes de texto?** 🞏 Sí 🞏 No

**Lugar de clase de CHARLAR: _________________________________________**

| **Información del Participante** | |
| --- | --- |
| **Me gustaría empezar con algunas preguntas de información personal. Por favor recuerde que todo lo que usted me diga será tratado confidencialmente. Por favor, escoja solamente una respuesta por pregunta.** | |
| 1. Sexo | 🞏 Masculino 🞏 Femenino 🞏 Otro |
| 1. Fecha de nacimiento | / /  Mes Día Año |
| 1. ¿Cuál es su raza o etnicidad?   (Por favor deja que se identifique los participantes ellos mismos; No lea la lista) | 🞏 Hispano/Latino/ 🞏 Blanco/Caucásico  Chicano/Mexicano 🞏 Negro/Africano Americano  🞏 Asiático 🞏 Isleño pacífico/Nativo de Hawái  🞏 Rehusó 🞏 Nativo Americano/o de Alaska  🞏 Otra (Especifique) |
| 1. ¿Cuál es su estado civil? | 🞏 Soltero/a 🞏 Divorciado/a  🞏 Casado 🞏 Viudo/a  🞏 Separado/a 🞏 Pareja  🞏 Rehusó |
| 1. ¿Cuántos años tiene usted de educación formal? | ____ Número de años  (Colegio completo o tiene GED = 12 años. Añade el número total de años en escuela, colegio, universidad o entrenamiento técnico) |
| 1. ¿Qué idioma habla usted? | 🞏 Sólo Español 🞏 Español e Inglés (bilingüe)  🞏 Sólo Ingles 🞏 Otro Idioma |
| 1. ¿Cuál idioma prefiere usar más? | 🞏 Español 🞏 No preferencia  🞏 Ingles 🞏 Otro |
| 1. ¿Dónde nació usted? | 🞏 Estados Unidos 🞏 México  🞏 Otro (Indique País) |
| 1. ¿Cuántos años ha vivido en los estados unidos? | 🞏 Menos de un año 🞏 6 – 10 años  🞏 1 – 5 años 🞏 Más de 10 años |
| 1. ¿Cuál es su estado de trabajo? | 🞏 Empleado/a 🞏 Jubilado/a  🞏 Desempleado/a 🞏 Sin habilidad de trabajar  🞏 Temporal 🞏 Estudiante  🞏 Ama de casa 🞏 Rehusó |
| 1. Muéstrele al participante la siguiente tabla y pregúntele si su ingreso familiar está dentro de la categoría A, B, C, D o E. Se presenta Anual, Mensual o Semanal según la preferencia del participante. | \|  \|  \| **Anualmente** \| **Mensualmente** \| **Semanalmente** \| \| --- \| --- \| --- \| --- \| --- \| \| A \| 🞏 \| Menos de 14,999 \| Menos de 1,250 \| Menos de 288 \| \| B \| 🞏 \| 15,000 a 19,999 \| 1,251 a 1,667 \| 288 a 385 \| \| C \| 🞏 \| 20,000 a 29,999 \| 1,668 a 2,499 \| 386 a 576 \| \| D \| 🞏 \| 30,000  a 59,999 \| 2,450 a 4,999 \| 577 a 1,153 \| \| E \| 🞏 \| 60,000 o más \| 5,000 o más \| 1,154 o más \|   🞏 Rehusó |
| 1. En los últimos 12 meses, ¿tuvo la preocupación de que se le iba a acabar el alimento antes de tener dinero para comprar más? | - A menudo - A veces - Nunca - No quiero contestar |
| 1. En los últimos 12 meses, ¿el alimento que compró no le rindió y no tuvo dinero para comprar más? | - A menudo - A veces - Nunca - No quiero contestar |
| 1. ¿Cuál es su situación de a vivienda hoy? | - Tengo vivienda - No tengo vivienda (quedarse con otros, en un hotel, en un refugio, viviendo afuera en la calle, en un automóvil o en un parque) - No quiero contestar |
| 1. ¿Se preocupa perder su vivienda? | - Sí - No - No quiero contestar |
| **Salud Mental** | |
| **La siguiente serie de preguntas le preguntará sobre sus niveles de estrés y salud mental, que se están convirtiendo en una rutina en todas las evaluaciones de salud. Estas son preguntas importantes que deben responderse porque se ha demostrado que el estrés es un factor de riesgo importante para las enfermedades del corazón, al igual que tener colesterol alto o presión arterial.** | |
| \| 1. En el último mes, ¿con qué frecuencia: \| Nunca \| Casi nunca \| De vez en cuando \| A menudo \| Muy a menudo \| \| --- \| --- \| --- \| --- \| --- \| --- \| \| 1. Se ha sentido incapaz de controlar las cosas importantes en su vida? \| 0 \| 1 \| 2 \| 3 \| 4 \| \| 1. Se ha sentido inseguro sobre su capacidad para manejar sus problemas personales? \| 0 \| 1 \| 2 \| 3 \| 4 \| \| 1. Ha sentido que las cosas no le salen bien? \| 0 \| 1 \| 2 \| 3 \| 4 \| \| 1. Ha sentido que los problemas se acumulan tanto que no puede superarlas? \| 0 \| 1 \| 2 \| 3 \| 4 \|  \| 1. Durante las últimas 2 semanas, ¿con qué frecuencia ha sentido molestias por los siguientes problemas? \| Nunca \| Varios días \| Más de la mitad de los días \| Casi todos los días \| \| --- \| --- \| --- \| --- \| --- \| \| G1. Sentirse nervioso/a, intranquilo/a o con los nervios de  punta \| 0 \| 1 \| 2 \| 3 \| \| G2. No poder dejar de preocuparse o no poder controlar la  preocupación \| 0 \| 1 \| 2 \| 3 \| \| G3. Preocuparse demasiado por diferentes cosas \| 0 \| 1 \| 2 \| 3 \| \| G4. Dificultad para relajarse \| 0 \| 1 \| 2 \| 3 \| \| G5. Estar tan inquieto/a que es difícil permanecer sentado/a tranquilamente \| 0 \| 1 \| 2 \| 3 \| \| G6. Molestarse o ponerse irritable fácilmente \| 0 \| 1 \| 2 \| 3 \| \| G7. Sentir miedo como si algo terrible pudiera pasar \| 0 \| 1 \| 2 \| 3 \|  \| 17. Durante las últimas 2 semanas, ¿con qué frecuencia ha sentido molestias por los siguientes problemas? \| Nunca \| Varios días \| Más de la mitad de los días \| Casi todos los días \| \| --- \| --- \| --- \| --- \| --- \| \| P1. Se ha sentido decaído(a), deprimido(a), o sin esperanzas \| 0 \| 1 \| 2 \| 3 \| \| P2. Poco interés o placer en hacer las cosas \| 0 \| 1 \| 2 \| 3 \| \| P3. Dificultad para dormir o permanecer dormido(a), o ha dormido demasiado \| 0 \| 1 \| 2 \| 3 \| \| P4. Se ha sentido cansado(a) o con poca energía \| 0 \| 1 \| 2 \| 3 \| \| P5. Con poco apetito o ha comido en exceso \| 0 \| 1 \| 2 \| 3 \| \| P6. Se ha sentido mal con usted mismo(a) – o que es un fracaso o que ha quedado mal con usted mismo(a) o con su familia \| 0 \| 1 \| 2 \| 3 \| \| P7. Ha tenido dificultad para concentrarse en cosas tales como leer el periódico o ver televisión \| 0 \| 1 \| 2 \| 3 \| \| P8. Se ha estado moviendo o hablando tan lento que otras personas podrían notarlo?, o por el contrario – ha estado tan inquieto(a) o agitado(a), que se ha estado moviendo mucho más de lo normal \| 0 \| 1 \| 2 \| 3 \| | |
| **Hábitos de Comer y Tomar** | |
| **Ahora, me gustaría preguntarle sobre sus hábitos de comer y beber. Acuerda que toda la información será tratado confidencialmente.** | |
| 1. En general, ¿qué tan seguido come vegetales (no incluya papas)? | Veces al día / semana / mes (MARQUE **UNO**)  🞏 Nunca  🞏 No sé |
| 1. En general, ¿qué tan seguido come fruta fresca (no incluya jugo)? | Veces al día / semana / mes (MARQUE **UNO**)  🞏 Nunca  🞏 No sé |
| 1. En general, ¿qué tipo de grasa utiliza más seguido la persona que cocina o prepara las comidas? Marque UNO**.** | 🞏 Manteca 🞏 Aceite de Maíz 🞏 No concina con grasa  🞏 Margarina 🞏 Aceite Vegetal 🞏 Otra (Especifique)  🞏 Manteca Animal 🞏 Aceite de Canola ___________________  🞏 Manteca Vegetal 🞏 Aceite de Oliva  (Crisco/Inca) 🞏 Aceite de Aerosol (Pam) |
| 1. En general, ¿qué tan seguido toma bebidas endulzadas con azúcar como jugo embotellado, soda (no dieta), café/té (con azúcar real) limonada, horchata, o bebidas de deporte como Gatorade? | Veces al día / semana / mes / año (MARQUE **UNO**)  🞏 Nunca  🞏 No sé |
| 1. ¿Qué tipo de leche toma regularmente? (MARQUE **UNO**) | 🞏 Leche entera 🞏 Leche condensada/evaporada  🞏 Leche de 2% 🞏 Leche de cabra  🞏 Leche de 1% 🞏 Leche de soya/arroz/almendra  🞏 Leche de 0% (sin grasa) 🞏 No toma leche |
| **Actividad Física** | |
| **Estamos interesados en saber de la actividad física que la gente hace. Piense en las actividades que usted hace APARTE DE su trabajo, en la casa y para ir de un sitio a otro.** | |
| 1. En una semana promedio, ¿cuántos días hace ejercicio? | 1 2 3 4 5 6 7 (MARQUE **UNO**) |
| 1. En un día promedio cuando hace ejercicio, ¿por cuánto tiempo hace ejercicio? | Horas por día O Minutos por día |
| **Piense del tiempo que usted dedicó a caminar en los últimos 7 días. Esto incluye trabajo en la casa, caminatas para ir de un sitio a otro, o cualquier otra caminata que usted hizo únicamente por recreación, deporte, ejercicio, o placer.** | |
| 1. Durante los últimos 7 días, ¿Cuántos días caminó usted por al menos 10 minutos continuos? | Días por semana  *Si “0-Cero” o no camina, avance al próxima sección* |
| 1. Usualmente, ¿cuánto tiempo camina usted en uno de eso días? *Seleccione horas o minutos.* | Horas por día O Minutos por día |
| **Alcohol y Drogas** | |
| **Las siguientes preguntas se tratan del uso de bebidas alcohólicas y marihuana.** | |
| 1. ¿Cuántas veces en el último año tuvo más de 5 bebidas en una sola sesión? | ____ veces |
| 1. ¿Actualmente fumas o consumes marihuana? | 🞏 No 🞏 Sí |
| **General** | |
| **Ahora le preguntaré sobre su salud en general.** | |
| 1. Usted dijera que su salud en general es: | 🞏 Excelente 🞏 Regular  🞏 Muy Buena 🞏 Mala  🞏 Buena 🞏 Rehusó |
| 1. ¿Tiene usted a alguien en su vida que pueda darle apoyo personal /social? Por ejemplo, alguien que le da consejos o charlas sobre sus metas personales. | 🞏 No 🞏 Sí |
| 1. ¿Cómo supo usted del programa CHARLAR? | 🞏 Clínica 🞏 Familiar  🞏 Promotor(a) 🞏 Amigo  🞏 Iglesia  🞏 Evento de la comunidad/presentación/Otra (Especifique):  ___________________________________________ |
| **Información del Cuidado de Salud** |  |
| **Ahora le preguntaré algunas preguntas sobre su cuidado de salud y su historia de salud.** | |
| 1. ¿Tiene un hogar médico, clínica, o medico(a) donde reciba su atención médica regularmente? | 🞏 No 🞏 Sí  Si sí:  Nombre de proveedor médico: _______________  Nombre de clínica: ____________________________  ¿Cuántas veces recibió atención médica ahí en el último año? ______ |
| 1. ¿Tiene usted alguno de los siguientes tipos de cobertura de salud? (Indique todos los que correspondan) | 🞏 Medicaid 🞏 Medicare  🞏 CICP/Otro programa de descuento 🞏 Ninguno  🞏 Seguro privado |
| **Historia de Salud** | |
| 1. ¿Fuma usted cigarrillos actualmente? | 🞏 No 🞏 Sí |
| Marque todos los que corresponden:   \|  \| ¿Le ha indicado un **médico, enfermero,** o **asistente del médico** que usted tiene: \|  \| Marque aquí si le recetaron un medicamento para la condición: \| Marque aquí si toma esta medicamento **diariamente**: \| Marque aquí si tomó el medicamento **hoy**: \| \| --- \| --- \| --- \| --- \| --- \| --- \| \| 1. **Diabetes** \| 🞏 No 🞏 Sí \| Si sí, \| 🞏 \| 🞏 \| 🞏 \| \| 1. **Presión arterial**   **alta** *(hipertensión)* \| 🞏 No 🞏 Sí \| Si sí, \| 🞏 \| 🞏 \| 🞏 \| \| 1. **Colesterol alto** *(high cholesterol)* \| 🞏 No 🞏 Sí \| Si sí, \| 🞏 \| 🞏 \| 🞏 \| \| 1. **Enfermedad de**   **Corazón** *(heart disease)* \| 🞏 No 🞏 Sí \| Si sí, \| 🞏 \| 🞏 \| 🞏 \| \| 1. **Derrame** *(stroke)* \| 🞏 No 🞏 Sí \| Si sí, \| 🞏 \| 🞏 \| 🞏 \| \| 1. **Ansiedad** \| 🞏 No 🞏 Sí \| Si sí, \| 🞏 \| 🞏 \| 🞏 \| \| 1. **Depresión** \| 🞏 No 🞏 Sí \| Si sí, \| 🞏 \| 🞏 \| 🞏 \| | |
| **Esto finaliza nuestra entrevista. Muchas gracias por su participación.** | |
| **Nombre de Entrevistador(a) (Letra de Molde Por Favor):______________________** | |
